# Patients’ perspectives on prescription cannabinoid therapies: a cross-sectional, exploratory, anonymous, one-time web-based survey among German patients

**DOI:** 10.3389/fmed.2023.1196160

**Published:** 2023-12-01

**Authors:** Jan Moritz Fischer, Farid I. Kandil, Ekaterina Katsarova, Laura Sophie Zager, Michael Jeitler, Felix Kugler, Franziska Fitzner, Vijayendra Murthy, Etienne Hanslian, Christoph Wendelmuth, Andreas Michalsen, Matthias Karst, Christian S. Kessler

**Affiliations:** ^1^Institute of Social Medicine, Epidemiology and Health Economy, Charité – Universitätsmedizin Berlin, Corporate Member of Freie Universität Berlin and Humboldt-Universität zu Berlin, Berlin, Germany; ^2^Department of Anesthesiology and Intensive Care Medicine, Pain Clinic, Hannover Medical School, Hannover, Germany; ^3^Faculty of Health, University of Technology Sydney, Ultimo, NSW, Australia; ^4^Praxis Wendelmuth, Potsdam, Germany; ^5^Department of Internal Medicine and Nature-based Therapies, Immanuel Hospital Berlin, Berlin, Germany

**Keywords:** cannabinoids, cannabis, survey, Germany, patients’ perspective, pain

## Abstract

**Introduction:**

Since cannabinoids were partially legalized as prescription medicines in Germany in 2017, they are mostly used when conventional therapies do not suffice. Ambiguities remain regarding use, benefits and risks. This web-based survey explored the perspectives of patients whose experiences are not well enough known to date.

**Methods:**

In an anonymous, exploratory, cross-sectional, one-time web-based observational study, participants receiving cannabinoid therapy on prescription documented aspects of their medical history, diagnoses, attitudes toward cannabinoids, physical symptoms, and emotional states. Participants completed the questionnaires twice here: first regarding the time of the survey and then, retrospectively, for the time before their cannabinoid therapy. Participants were recruited in a stratified manner in three German federal states.

**Results:**

*N* = 216 participants (48.1% female, aged 51.8 ± 14.0) completed the survey, most of which (72%, *n* = 155) reported pain as their main reason for cannabinoid therapy. When comparing the current state with the retrospectively assessed state, participants reported greater satisfaction with their overall medical therapy (TSQM II: +47.9 ± 36.5, *p* < 0.001); improved well-being (WHO-5: +7.8 ± 5.9, *p* < 0.001) and fewer problems in PROMIS subscales (all *p* < 0.001). Patients suffering primarily from pain (72%, *n* = 155) reported a reduction of daily pain (NRS: −3.2 ± 2.0, *p* < 0.001), while participants suffering mainly from spasticity (8%, *n* = 17) stated decreased muscle spasticity (MSSS: −1.5 ± 0.6, *p* < 0.001) and better physical mobility (−0.8 ± 0.8, *p* < 0.001). Data suggests clinically relevant effects for most scores. Participants’ attitudes toward cannabinoids (on a 5-point scale) improved (+1.1 ± 1.1, *p* < 0.001). Most patients (*n* = 146, 69%) did not report major difficulties with the cannabinoid prescription process, while (*n* = 27; 19%) had their cannabinoid therapy changed due to side effects.

**Discussion:**

Most participants experienced their therapy with cannabinoids as more effective than their previous therapy. There are extensive limitations to this cross-sectional study: the originally intended representativeness of the dataset was not reached, partly due to the SARS-CoV-2 pandemic; the sample has a larger proportion of privately insured and self-paying patients. Results does not suggest that cannabinoid patients belong to a particular clientele. Effect sizes observed for pain reduction, quality of life, social participation, and other outcomes suggest a therapeutic potential, particularly in the treatment of chronic pain.

## 1 Introduction

Medical prescription cannabinoids were partially legalized in Germany in March 2017 and are since being prescribed increasingly by physicians from different specialties. However, controversy surrounding their medical use, benefits, risks, and problems in clinical practice and beyond persists. This includes medical, health economic, political, and societal concerns (1). Additionally, the current German government coalition has initiated a legislative process to partially legalize/decriminalize cannabis use for the general population, influencing national-level political debates on the use of cannabinoids in medical contexts (2, 3).

Since 2017, physicians in Germany can prescribe a range of medical cannabinoids under German law (4, 5). According to an official surveillance study (6), the most common reasons for cannabinoid prescriptions in 2017–2022 were pain 76.4% (*n* = 12842 of a total *N* = 16809), neoplasia 14.5% (*n* = 2434), spasticity 9.6% (1607), multiple sclerosis 5,9% (*n* = 989), anorexia/weight loss 5.1% (*n* = 852), depression 2.8% (*n* = 471), nausea/vomiting 2.2% (*n* = 376), migraine 2.0% (*n* = 332), and attention deficit hyperactivity disorder (ADHD) 1.0% (*n* = 163). Cannabinoids are also commonly prescribed for pain in Australia (7), the United Kingdom (8), France (9), and the United States (10, 11).

Until March 2022, physicians prescribing cannabinoids on narcotic prescription at the expense of German statutory health insurance carriers were required to participate in a national online surveillance survey (6), which collected data on indications, perceived clinical efficacy, observed adverse effects, details of prescribed medications, dosages, concomitant therapies, and so on. In this survey, all data were collected exclusively from the prescribing physicians and reflect only their perspective. In the absence of class Ia, Ib and II evidence from controlled clinical trials and systematic reviews/meta-analyses, the results and findings from this important survey has formed the basis for many of the expert medical and political debates on the regulation of prescription cannabinoids in Germany.

All licensed physicians in Germany can prescribe THC-containing cannabinoids on narcotic prescriptions (these are special prescriptions that can be ordered from the German Federal Institute for Drugs and Medical Devices (BfArM) and are personalized to the respective physician). No special permission is required from the BfArM to prescribe cannabinoids.

The prescription duration depends on the specific dosages used and the maximum prescription quantities for the respective cannabinoid drugs. For example, for medical cannabis flowers, a maximum of 100 g may be prescribed per one narcotic prescription. Usually, the prescription quantity corresponds to the requirement for one month. However, it is possible to prescribe higher doses, both in terms of the period of use and in terms of the maximum monthly prescription quantity. In this case, the prescription must be specially marked (with an “A”).

Both finished medicines (e.g., nabiximols, nabilone) and magistral prescriptions, such as cannabinoid extracts and medical cannabis flowers, are available in different concentrations of THC and CBD. In Germany, only cannabinoids with a minimum concentration of THC of >0.2% can be medically prescribed (cannabinoids with a THC content below 0.2% are not (yet) subject to prescription and are usually on the market as dietary supplements). Different galenics can be prescribed, e.g., products for oral use or for inhalation.

The prescription related decisions are made relying on both the experience of the prescriber and on previous experiences of the patient. When it comes to reimbursement (for which a request to the health insurance company is still necessary), the greatest acceptance on the part of German health insurance carriers is for chronic (nerve) pain, while specific pain syndromes, such as rheumatic-inflammatory pain, migraine or psychiatric diagnoses (e.g., PTSD), are oftentimes not supported. The overall rejection rate is about 40%. Regarding the cannabinoid products, the funding agencies primarily support finished medicines and extracts while cannabis flowers are only supported in exceptional cases.

There have been few major clinical research projects on cannabinoids funded by the public sector, industry, or philanthropy, in the nearly six years since the law was changed in Germany. The lack of effective funding opportunities has led to a peculiar situation with regard to cannabinoids in medicine, where, on the one hand, there is much controversy about this topic in the medical profession and society, but, on the other hand, it is quite difficult in Germany to generate the clinical evidence that is often demanded – a kind of “cannabis dilemma 2.0” (12).

The aim of this study was to investigate patients’ experiences and perspectives regarding prescription cannabinoid treatments in outpatient settings in Germany.

## 2 Materials and methods

### 2.1 Study design

The methods of this study have been described in detail in a previous protocol publication (13).

In brief, this cross-sectional study was conducted as an anonymous, one-time, exploratory, web-based survey of prescription cannabinoid patients between May 31, 2021, and June 2022 by the Institute of Social Medicine, Epidemiology and Health Economy of Charité – Universitätsmedizin Berlin, Outpatient Clinic for Integrative Medicine at Immanuel Hospital Berlin, Germany.

The study was conducted in the three German states of Berlin, Lower Saxony and Brandenburg and included both rural and urban populations. Recruitment with the assistance of physicians from different specialties ensured that only patients who were actually treated with prescription cannabinoids could participate. Special attention was paid to anonymity to reduce treatment provider influence and stigma. All of these measures were chosen to reduce selection and response bias.

In the form of mixed-methods approach, information from qualitative interviews helped to refine the quantitative methods, allowing a broader range of relevant aspects of cannabinoid therapies to be captured. As a separate project, these qualitative interviews with 32 outpatients suffering from chronic pain and treated with cannabinoids at Hannover Medical School (MHH) for a minimum of 6 months preceded the study (MHH Ethics Vote 8391_BO-K_2019). Following the methods described in detail in Fischer et al. (13), all participants were interviewed by the same researcher (FF) who was also responsible for transcribing and analyzing the interviews. The insights into cannabinoid therapy gained from the patient perspective prompted us to ask participants about their previous experiences with relaxation techniques or psychotherapy, pre-existing or symptom-related psychological trauma, their personal opinions about cannabinoids, and different experiences with various cannabis-based medications. However, a comprehensive account of the analysis of these qualitative interviews is beyond the scope of this study and will be presented in a separate publication.

### 2.2 Setting

The original intent of this study was to draw a representative sample of patients treated with cannabinoids in the two German federal states of Berlin (city state) and Lower Saxony. SARS-CoV-2 pandemic and its contact regulations reduced the number of physical visits of patients to their doctor’s practices and lowered thus the recruitment rate. To counter that, the state of Brandenburg was added in an amendment on January 20, 2022. Physicians with specialist qualifications in anesthesiology, general and family medicine, neurology, and internal medicine, who hold a statutory health insurance contract in one of the federal states, were initially contacted in a stratified procedure, as well as outpatient tertiary centers. These specialist groups were selected because, according to the aforementioned surveillance survey, they account for 88.6% of cannabinoid prescriptions in Germany (6). Physicians were contacted by e-mail or fax to invite their patients treated with cannabinoids to participate in this anonymous web-based cross-sectional survey. In a second step, the same physicians were also contacted by telephone, in random order and in quotas that reflected the proportion of cannabinoid prescriptions for each specialty group, because we did not expect a sufficient response in the first step. Calls were to be made until the target number of participants was reached. Due to the late amendment toward the end of the recruitment phase, this second step could not be implemented in the state of Brandenburg.

### 2.3 Participants

As is common for exploratory studies, the sample size was not calculated based on previously described effect sizes but set to *n* = 300 owing to resources. The target sample size of *n* = 300, however, is sufficient to detect (descriptive) effects with an effect size of Cohen’s *d* > 0.17 and thus to distinguish large (*d* > 0.80), moderate (*d* between 0.50 and 0.80), small (*d* between 0.20 and 0.50) and negligible effects (*d* < 0.20) from one another (14).

**Figure 1:**
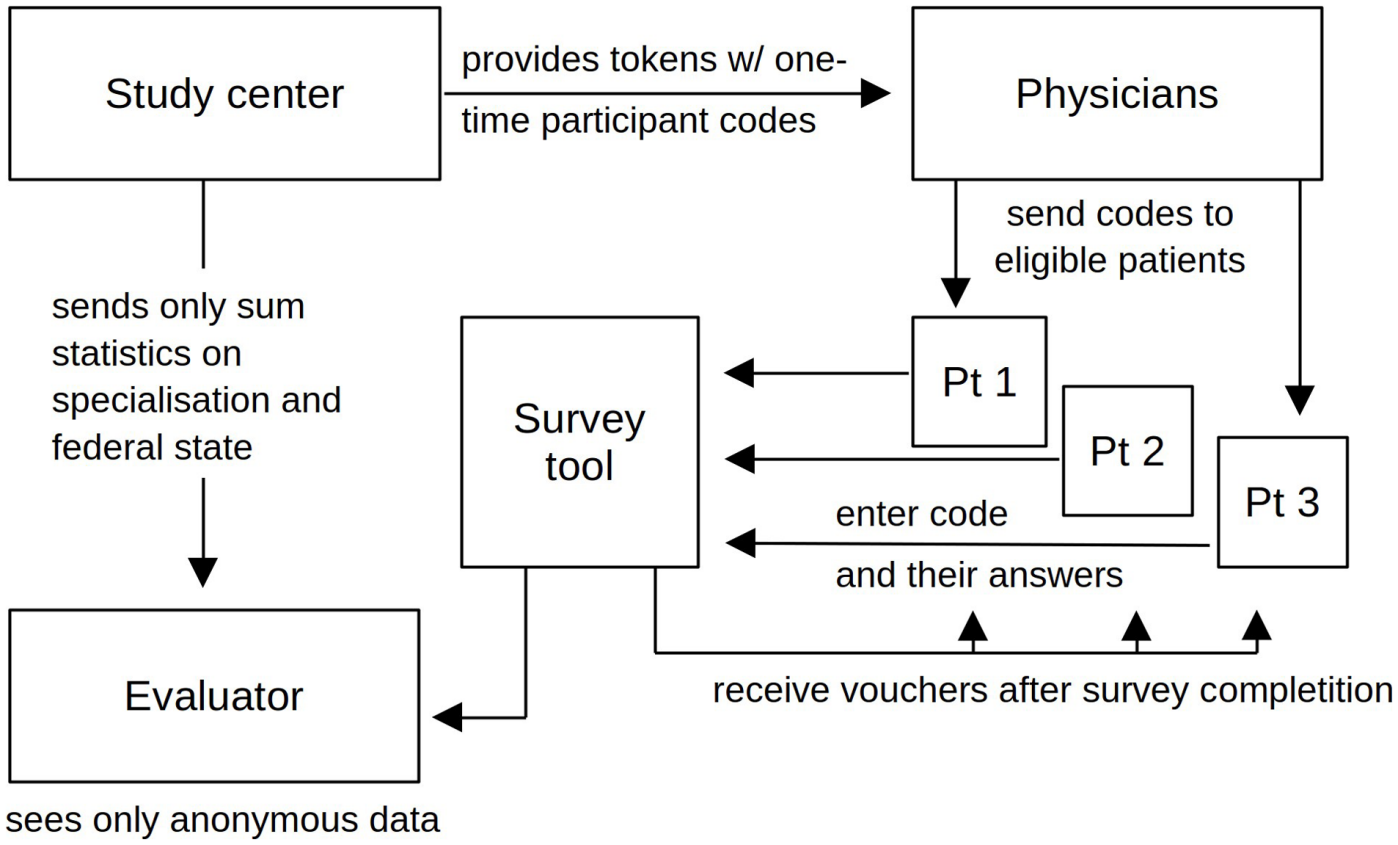
Information flow ensuring participant anonymity.

Interested physicians received informational materials and survey access codes to distribute to their cannabinoid patients, including those who had completed or discontinued such therapies in the previous 12 months. With information and access code, patients could later decide at home whether or not to participate and provide all information discreetly and anonymously. The cooperating physicians were compensated for their time and effort in inviting patients to the study with 50 Euros per patient who eventually participated (13). Each unique participation login code could only be used once to complete the web-based survey. Thus, the study center did not receive personalized or pseudonymized participant data, and the prescribing physicians did not have access to study data (Figure 1). Active recruitment ended on May 31, 2022; the last participant completed this survey on June 12, 2022. More details can be found in the protocol publication (13).

### 2.4 Outcome parameters and variables

**Figure 2:**
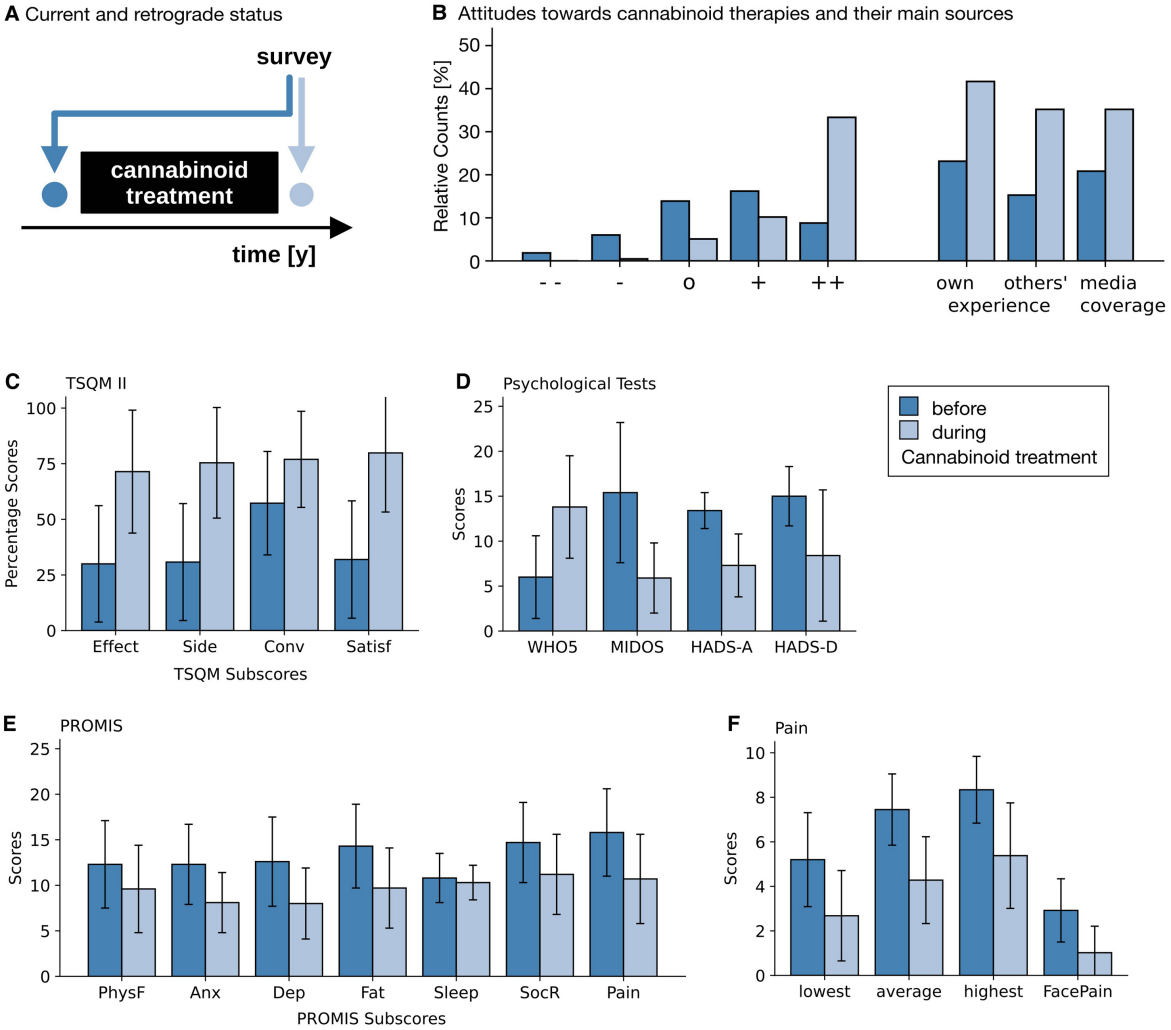
Results of questionnaires and NRSs. **(A)** Temporal aspects of the survey. During the survey, attitudes and questionnaires were answered twice. Once for the then current point in time (light blue) and once for the recalled time point prior to the commencement of the cannabinoid therapy. **(B)** Attitudes toward cannabinoid therapy (left), with ++, +, o, −, and - - indicating very positive, rather positive, neutral, rather negative and very negative attitudes; as well as the base for that attitude (right) before the start of the therapy (dark blue) and at the time of the survey (light blue). **(C)** Satisfaction with the current therapy (Treatment Satisfaction Questionnaire for Medication, version II, TSQM II, for all 216 patients), with its sub scores effectiveness (effect), side effects (side), convenience (conv) and overall satisfaction (satisf). **(D)** Psychological questionnaires for quality of life (5-item World Health Organization Well-Being Index, WHO-5, for all 216 patients), distressing symptoms (Minimal Documentation System on Distressing Symptoms, MIDOS, for the 32 patients with weight-loss and nausea), anxiety (Hospital Anxiety and Depression Scale, HADS-A) and depression (Hospital Anxiety and Depression Scale, HADS-D, both for the 8 patients with either depression or anxiety symptoms). **(E)** PROMIS (Patient Reported Outcome Measurement Information System) scores (for all 216 patients) for physical functioning (PhysF), anxiety (Anx), depression (Dep), fatigue (Fat), Sleep Disturbances (Sleep), constraints of fulfilling one’s social role (SocR) and overall pain (Pain). **(F)** Pain Scales (for the 155 patients indicating pain as their primary reason for their cannabinoid therapies). Patients indicated the strength of minimal, average and maximal pain during the day on a 0–10 Numerical Rating Scale (NRS) and on a 6-point graphical rating scale displaying faces (GRS). Light blue bars show the current self-assessment, dark blue bars indicate the status prior to the start of the cannabinoid therapy. Whiskers indicate Standard Deviations (SD). Note that an improvement is associated with higher values in all TSQM II subscales and in the WHO-5 score, and with lower values in all other scores.

In this survey, subjects were asked to complete all questionnaires and questions regarding their cannabinoid therapy twice in the same session: once for the current time point (i.e., at the date of the survey participation), and then a second time for the time point prior to the commencement of the cannabinoid therapy, by self-recalling that period (*cf.* Figure 2A) (13).

All participants completed the *Treatment Satisfaction Questionnaire for Medication* (TSQM, version II) (15, 16), the *WHO-5* quality of life questionnaire (17), the *Patient Reported Outcome Measurement Information Systems* (PROMIS-29), numeric rating scales (NRS) for the daily average, lowest and strongest pain (18, 19), and the *Faces Pain Scale* (20, 21). In addition, the following validated questionnaires were displayed to the patients suffering from the respective conditions: the *Hospital Anxiety and Depression Scale* (HADS) (22–25) for patients with symptoms of depression or anxiety, the *Multiple Sclerosis Spasticity Scale 88* (MSSS-88) (26) for participants with multiple sclerosis, the *Anorexia Nervosa Inventory for Self-Rating* (ANIS) (27) and the *Minimal Documentation System on Distressing Symptoms* (MIDOS2) for participants with anorexia-related and non-anorexia-mediated weight loss and nausea/vomiting (28), respectively, and the *Adult ADHD Self-Report Scale* (ASRS) (29, 30) for participants with ADHD-associated symptoms. Licenses were obtained for questionnaires prior to the commencement of the study, wherever required.

Sociodemographic data were collected (age, occupational status, highest level of education, relevant main diagnoses, previous and current other therapies, medication, side effects) including previous experiences with pre-existing or symptom-related psychological trauma, and relaxation techniques or psychotherapy, patients’ personal opinions about cannabinoids, and varying experiences with different cannabis-based medications. These included the exact cannabis preparation, dose, mode of application, adverse effects, and reasons for pre-prescription use of cannabinoids (if applicable). The duration of cannabis therapy was asked, as well as the symptoms for which it was prescribed. The most common symptoms (6) of pain, spasticity, weight loss, nausea/vomiting, depression, attention-deficit/hyperactivity disorder (ADHD), and a free-text field were available as possible answers. Underlying conditions were asked as free text and ICD number.

Participants were also asked about details of the cannabinoid prescription process, particularly if there were any problems in obtaining the cannabinoid medication. Response options ranged from “very major problems” (+2) to “no problems at all” (−2). If problems were reported, participants were asked at which point in the health care system those had occurred (family doctor, specialist, health insurance company, pharmacy, elsewhere [free text]). Health insurance status was queried (as between (i) statutory health insurance with or without specialized supplementary private insurances, e.g., for certain dental treatment or eyewear, (ii) complete private health insurance, (iii) none at all or (iv) something else [free text]. For details about German health insurance see reference (31)). General attitudes toward cannabinoids, before and after the start of cannabinoid therapy, were rated on a 5-point Likert scale, ranging from very positive (+2) to very negative (−2). It was asked what these attitudes were based on (e.g. experiences, media reports, personal experiences, free text). Changes in cannabinoid medication were asked, as were the reasons for any changes (insufficient effect, side effects, interactions, therapy no longer necessary, reason not known, free text). In addition, it was asked from whom the idea for cannabinoid therapy originally came (family, friends/acquaintances, treating physician, patient him/herself, free text). (For details, see reference 13).

### 2.5 Data sources and measurement

The survey (Supplement 1) was implemented using Lime Survey software (Lime Survey GmbH, Hamburg, Germany, v 5.4). Informed consent was declared anonymously online and was a prerequisite for data entry along with the login code. Software and data were stored on a server hosted and secured by Charité – Universitätsmedizin Berlin. The study was approved by the Charité ethics committee on December 16, 2020 (EA1/327/20) and registered at the German Register of Clinical Studies (DRKS00023344).

### 2.6 Statistical methods

Data of this exploratory, cross-sectional study were evaluated primarily using descriptive statistics. Absolute and relative frequencies and parametrical descriptive statistics were calculated along with t-tests using Python (v 3.9) within a non-confirmatory context. As common in exploratory studies, an alpha of 0.05 was applied throughout, without any (Bonferroni) correction for multiple testing. As a result, all *p* < 0.05 are considered “exploratorily significant,” meaning that none of these tests are considered to be confirmatory of any hypothesis by themselves, but interesting enough to be tested more thoroughly in future confirmatory studies. To compare the effects of cannabinoid therapy between the various tests applied here and with previous publications, results are also presented as effect sizes (Cohen’s d). Individual t-tests were applied to assess (on a strictly exploratory level) whether within-group changes constitute a substantial improvement. This assessment relies on both significance levels and effect sizes (Cohen’s d).

Furthermore, as a sensitivity analysis, results for the main tests for pain (NRS), treatment satisfaction (TSQM II) and quality of life (WHO-5) were tested as potential confounders by extending the t-test to mixed-design ANOVA models. Interaction terms indicate a putative significance of the confounders. We applied this analysis for the following possible confounders: sex, age, income, prior attitude toward cannabinoid therapies, prior own experience with cannabis, treatment duration, initiative to start the treatment, insurance type, reimbursement, type of medical cannabinoid, traumatic experiences, and additional therapies.

## 3 Results

### 3.1 Contacted medical practices

First, a total of 11,744 physicians (1,700 in Berlin, 8,003 in Lower Saxony, 2,041 in Brandenburg) were contacted by e-mail and fax. In the second step, these physicians were contacted again by telephone in a randomized order, so that the number of physicians in all three federal states and in the different medical specialties was contacted in proportion to their relative share of patients (6) until the quotas were reached. 358 calls were made to physicians in Berlin and 1,181 calls to physicians in Lower Saxony. As mentioned in the methods section, the second step had to be omitted in Brandenburg due to pandemic-related time constraints. Of all physicians contacted, 43 physicians indicated that they treated patients with cannabinoids and were interested in inviting them to participate in the study. 34 physicians finally motivated patients to participate in the web-based survey (13 in Berlin, 17 in Lower Saxony and 4 in Brandenburg). 9 were anesthesiologists, 17 were general practitioners or family doctors, 4 were neurologists and 4 were specialists in internal medicine.

### 3.2 Participants

**Figure 3:**
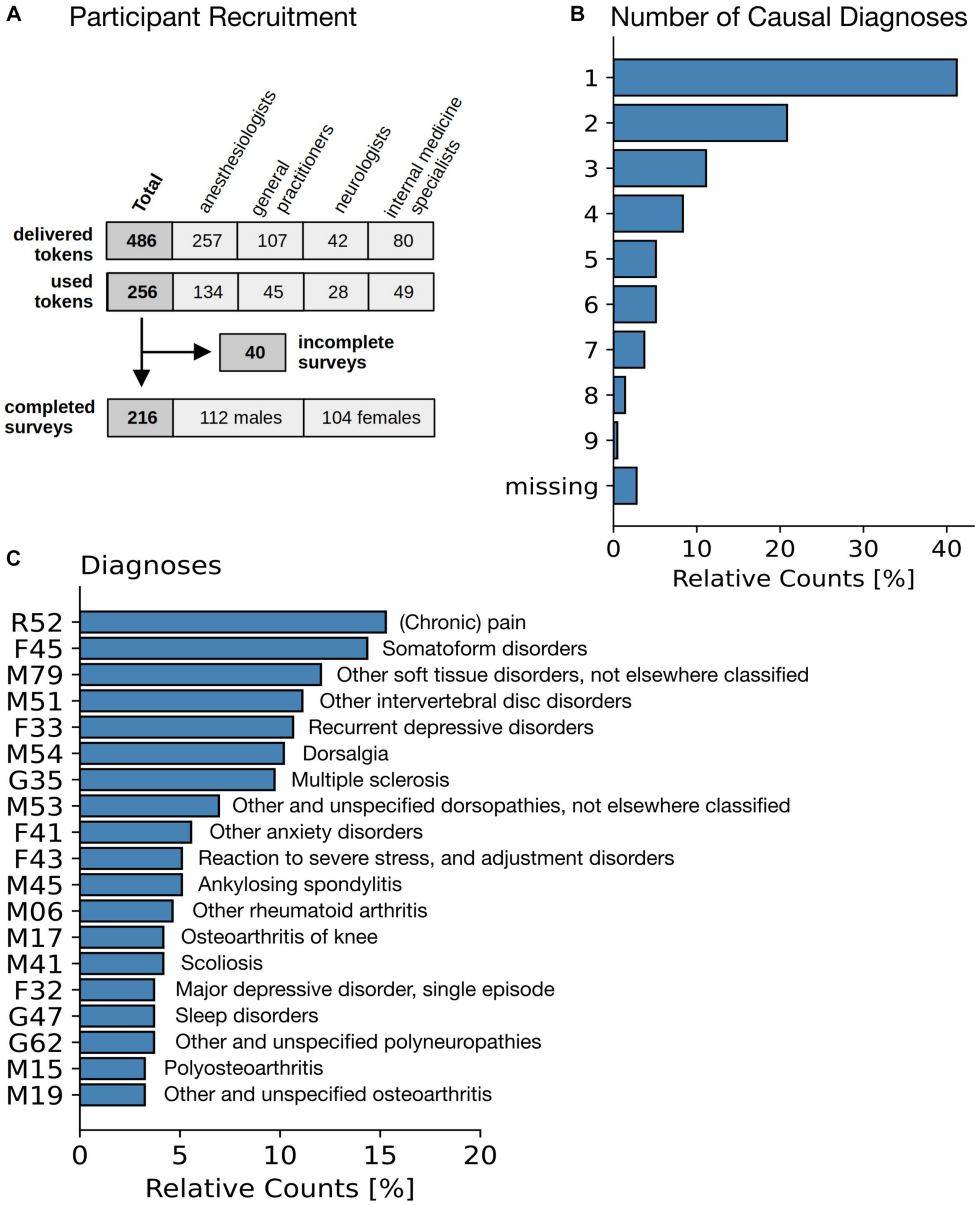
Patient characteristics. **(A)** Flowchart of participants. **(B)** Number of individual diagnoses stated as a justification for the cannabinoid therapy. **(C)** Diagnosis (International Classification of Diseases ICD-10) for the most common diagnoses leading to a treatment with cannabinoids.

A total of 486 invitations with individual login codes were sent to these physicians (Figure 3A), of which 256 were activated by the participants to start the survey. 132 (of 257) by patients of anesthesiologists, 45 (of 107) by patients of general practitioners, 28 (of 42) by patients of neurologists, and 49 (of 80) by patients of internists. Of the 256 surveys started, 40 were discontinued after the first few questions and were therefore excluded from the analysis (because the study was conducted anonymously, we do not have details about their reasons for discontinuing the survey. However, we speculate that they mistakenly expected to be able to resume the survey later.) The remainder, a total of *n* = 216 participants, 112 male and 104 female, completed the survey.

The originally planned number of *n* = 300 participants could not be reached until the end of May 2022, despite intensive recruitment efforts and an extension of the recruitment period. When we contacted physician practices, we repeatedly received feedback that there was no capacity to support the study due to the pandemic. We believe that significantly more physicians would have invited their cannabinoid patients to participate under pre-pandemic conditions. Due to limited human resources and study budget, the study was stopped at *n* = 216 completed surveys included in the final data analysis. However, the reached participant number of *n* = 216 is still sufficient to detect effects with effect sizes of Cohen’s *d* > 0.195, so that the distinction between the main effect sizes (small, moderate, large) is still ensured (14).

The mean age of cannabinoid patients was 51.8 (± 14.0) years (for details see Table 1). Participants reported to have been on prescription cannabinoid therapy on average for 2.4 ± 1.4 years. The highest educational attainment reported by *n* = 50 (23%) was high school graduation, *n* = 58 (27%) was an apprenticeship, *n* = 34 (16%) was a college degree, *n* = 56 (26%) was a university degree. *N* = 82 (38%) of the participants were working, *n* = 85 (39%) retired, and *n* = 37 (17%) permanently ill (other responses: *n* = 12, 6%).

**Table 1:** Baseline characteristics.

		All participants		Male		Female	
Parameter	Parameter value	*M*	*SD*	*M*	*SD*	*M*	*SD*
Height [cm]		173.6	9.5	180.0	7.0	166.8	6.6
Weight before therapy [kg]		80.0	21.6	87.4	22.0	72.0	18.1
Weight, current [kg]		78.4	18.3	84.9	18.1	71.3	15.8
BMI before therapy [kg/m^2^]		26.4	6.6	26.9	6.5	25.9	6.6
BMI, current [kg/m^2^]		25.9	5.5	26.2	5.4	25.7	5.6
Age [y]		51.8	14.0	47.9	13.3	56.0	13.5
		*n*	%	*n*	%	*n*	%
Age group	18–30	17	7.9	12	10.7	5	4.8
	31–50	83	38.4	56	50.0	27	26.0
	51–65	79	36.6	31	27.7	48	46.2
	66 +	37	17.1	13	11.6	24	23.1
Sex		216	100.0	112	51.9	104	48.1
Relationship status	Single	46	21.3	28	25.0	18	17.3
	Partnered	35	16.2	21	18.8	14	13.5
	Married	102	47.2	52	46.4	50	48.1
	Divorced	25	11.6	9	8.0	16	15.4
	Widowed	4	1.9	0	0.0	4	3.8
	Other	4	1.9	2	1.8	2	1.9
Persons living in household	None	7	3.2	3	2.7	4	3.8
(besides participant)	1	75	34.7	44	39.3	31	29.8
	2	117	54.2	57	50.9	60	57.7
	3	10	4.6	4	3.6	6	5.8
	≥ 4	7	3.2	4	3.6	3	2.9
Children in household	None	163	75.5	78	69.6	85	81.7
	1	32	14.8	22	19.6	10	9.6
	2	15	6.9	8	7.1	7	6.7
	≥ 3	6	2.8	4	3.6	2	1.9
Highest educational level	High school	50	23.1	28	25.0	22	21.2
	College diploma	34	15.7	20	17.9	14	13.5
	Apprenticeship	58	26.9	26	23.2	32	30.8
	University	56	25.9	30	26.8	26	25.0
	Other/missing	18	8.3	8	7.1	10	9.6
Professional status	Employed	82	38.0	50	44.6	32	30.8
	Retired	85	39.4	39	34.8	46	44.2
	Long-term sick leave	37	17.1	17	15.2	20	19.2
	Unemployed	2	0.9	2	1.8	0	0.0
	Other	10	4.6	4	3.6	6	5.8
Monthly income (participant)	<1,000€	43	19.9	20	17.9	23	22.1
	1,001€ – 1500€	50	23.1	17	15.2	33	31.7
	1,501€ – 2000€	40	18.5	22	19.6	18	17.3
	2001€ – 3000€	43	19.9	27	24.1	16	15.4
	3,001€ – 4000€	19	8.8	13	11.6	6	5.8
	> 4,000€	12	5.6	8	7.1	4	3.8
	Missing	9	4.2	5	4.5	4	3.8
Monthly income (household)	<1,500€	45	20.8	22	19.6	23	22.1
	1,501€ – 2000€	21	9.7	12	10.7	9	8.7
	2001€ – 3000€	42	19.4	21	18.8	21	20.2
	3,001€ – 4500€	53	24.5	31	27.7	22	21.2
	4,501–6,000€	28	13.0	11	9.8	17	16.3
	>6,000€	14	6.5	8	7.1	6	5.8
	Missing	13	6.0	7	6.3	6	5.8

M = mean, SD = standard deviation, n = count, % = percentage, BMI = Body Mass Index.

### 3.3 Diagnoses

The most common symptoms based on which prescription was made were chronic pain (*n* = 155, 72%), spasticity (*n* = 17, 8%), depression (*n* = 8, 4%), ADHD (*n* = 4, 2%) (remainder: *n* = 32, 15%). This is in-line with numbers reported in a previous German study (6). Additionally, participants were asked to enter all diagnoses known to them from their medical records in free text and, if available, along with the corresponding ICD-10 codes (32). Missing ICD codes were post-coded by study staff based on participants’ free text entries and validated by the study physician. Participants were also asked which of these diagnoses were primarily to be alleviated by cannabinoid therapy. In most cases one (*n* = 89, 41%), two (*n* = 45, 21%) or three (*n* = 24, 11%) different diagnoses were given (see Figure 3B). The most frequent diagnoses by International Classification of Diseases 10th revision (ICD-10) were (Figure 3C) chronic pain (R52), somatoform disorders (F45), other soft tissue disorders, not elsewhere classified (M79), and other intervertebral disc disorders, incl. Thoracic, thoracolumbar and lumbosacral disc disorders (M51) (*cf.* Figure 3C).

### 3.4 Attitudes toward cannabis and cannabinoids

Before presenting “pre-post” comparisons (current health status compared to self-reported health status before cannabinoid therapy in retrospect) below, we explicitly point out to the fact that these were calculated from a one-time cross-sectional survey (*cf.* Figure 2A). The presented exploratory significance levels and effect sizes must therefore be interpreted with appropriate caution and due restraint and no causal attributions are being derived from this exploratory observational data set (*cf.* the Discussion section).

Most patients reported to have had a neutral or mildly positive opinion of cannabinoids before starting cannabinoid therapy (Figure 2B). This was based in roughly equal measure on their own experiences with cannabis, on statements by their friends and family, and on media coverage. After the start of their cannabinoid treatment, most participants (*n* = 204, 94%) reported a positive attitude toward cannabinoids. Most of them based this on their own experiences and those of their friends. The shift toward more positive attitudes (on a 5-point Likert scale from −2 to +2) was even greater among participants without prior experience (+0.33 ± 1.11 to +1.61 ± 0.65) than for patients with prior experience (+0.81 ± 0.91 to +1.73 ± 0.51).

Previous experience with cannabis prior to medically prescribed cannabinoids was reported by *n* = 93 (43%) of participants, of which *n* = 64 (30%) reported having had taken it for medical reasons, *n* = 38 (18%) for recreational purposes, *n* = 28 (13%) out of curiosity, *n* = 35 (17%) in peer-group settings, *n* = 2 (1%) reported having tried cannabis due to peer pressure and none for religious reasons (multiple entries possible).

### 3.5 Experiences regarding the prescription process

Participants reported that the current cannabinoid prescription process had been suggested first by the participants themselves in *n* = 101 (47%) of cases, in *n* = 86 (40%) by the treating physician, and in *n* = 11 (5%) and *n* = 8 (4%) by friends and family members, respectively. The majority of participants (*n* = 175; 81%) had statutory health insurance coverage, and *n* = 34 (16%) were privately insured, which roughly corresponds to the average in the German population (31, 33). Of those holding private insurance, *n* = 31 out of 34 (91%) were reimbursed by their private health care insurer for the cost of cannabinoid prescriptions. In contrast, only 133 out of 175 (76%) of those with statutory health insurance were reimbursed by their health insurer. Accordingly, the remaining 42 of 175 (24%) paid for their cannabinoid medications out of pocket.

*N* = 70 (31%) of participants reported encountering barriers to accessing cannabinoid treatment (*n* = 39, 18% minor problems and *n* = 31, 13% major problems). The problems were primarily with the health insurance company (*n* = 56; 26%). *N* = 24 (11%) were with the specialist, *n* = 17 (8%) with the primary care physician, and *n* = 8 (4%) with the pharmacy. *N* = 146 (69%) of the participants indicated that they did not experience any problems and found it easy or very easy to get prescription cannabinoids.

### 3.6 Cannabinoid preparations and changes in therapy

Cannabinoids were prescribed in the form of cannabis flowers (*n* = 97; 45%), cannabis extracts (*n* = 52; 24%), dronabinol (equivalent to marinol; *n* = 43; 20%), and nabiximols (*n* = 27; 13%; multiple answers permitted). Of the *n* = 149 participants with a prescription for cannabis flower or extracts, *n* = 16 (11%) took it frequently or always with tobacco, *n* = 13 (7%) occasionally.

On average, participants had received prescription cannabinoids for 1–2 years at the time of the survey. During their therapy, most of the participants’ cannabinoid therapy was modified at least once (*n* = 139 out of *n* = 216, i.e., 64%). In those *n* = 139 participants the dose was either increased (*n* = 83, 60%) or reduced (*n* = 21, 15%), the medical cannabis cultivar was changed (*n* = 57, 41%), the mode of application, e.g., from drinking tea to vaporization (*n* = 9, 6%), and/or the medicinal product was changed, e.g., from nabiximols to dronabinol (*n* = 35, 25%).

The reason for these changes were in *n* = 100/139 cases (72%) a desired change in the effectiveness of the drug, in *n* = 27 (19%) occurrence of adverse side effects and in *n* = 5 (4%) interactions with other drugs. Among patient-reported adverse events, the most commonly reported were dry mouth (*n* = 14, 7%), attention-deficit (*n* = 11; 5%), fatigue (*n* = 10, 5%), dizziness (*n* = 10, 5%), and somnolence (*n* = 9, 4%).

### 3.7 Questionnaires: perceived changes in symptoms and well-being

The results for the validated questionnaires on how participants’ physical and mental symptoms and their perceived quality of life subjectively changed during cannabinoid therapy are shown in Table 2.

**Table 2:** Comparison between the current health status to the self-recalled health status prior to cannabinoid therapy commencement.

				Before therapy		Current status		Change		*t*-test		
Scale	Subscale	Sex	*n*	*M*	*SD*	*M*	*SD*	*M*	*SD*	*T*	*p*	*d*
Attitude toward Cannabis		All	216	0.5	1.05	1.7	0.60	1.1	1.13	14.64	<0.001	>1.00
		M	112	0.6	1.04	1.7	0.59	1.1	1.09	10.66	<0.001	>1.00
		F	104	0.5	1.07	1.7	0.60	1.2	1.18	10.03	<0.001	>1.00
TSQM II	Effectiveness	All	216	30.0	26.16	71.4	27.63	41.5	35.80	17.02	<0.001	>1.00
		M	112	32.2	27.47	77.0	25.45	44.8	32.96	14.39	<0.001	>1.00
		F	104	27.6	24.59	65.4	28.72	37.8	38.46	10.03	<0.001	>1.00
	Side effects	All	216	30.8	26.29	75.4	24.85	42.9	33.07	13.16	<0.001	>1.00
		M	112	31.0	28.23	79.6	22.50	45.4	36.03	8.91	<0.001	>1.00
		F	104	30.6	24.40	71.0	26.58	40.5	30.17	9.77	<0.001	>1.00
	Convenience	All	216	57.3	23.24	77.0	21.60	19.7	30.78	9.41	<0.001	0.88
		M	112	59.7	24.02	74.7	20.68	15.0	30.94	5.13	<0.001	0.67
		F	104	54.6	22.20	79.4	22.40	24.8	29.94	8.43	<0.001	>1.00
	Overall	All	216	31.9	26.37	79.8	26.56	47.9	36.47	19.30	<0.001	>1.00
	Satisfaction	M	112	32.0	27.61	82.5	24.04	50.6	36.38	14.71	<0.001	>1.00
		F	104	31.9	25.10	77.0	28.88	45.0	36.53	12.57	<0.001	>1.00
WHO 5	(Global)	All	216	6.0	4.61	13.8	5.75	7.8	5.88	19.47	<0.001	>1.00
		M	112	6.3	4.63	14.9	5.77	8.6	6.11	14.91	<0.001	>1.00
		F	104	5.6	4.59	12.5	5.47	6.9	5.52	12.77	<0.001	>1.00
PROMIS	Physical	All	216	12.4	4.80	9.6	4.81	−2.8	3.80	10.66	<0.001	0.57
	functioning *	M	112	11.5	5.11	8.3	4.84	−3.2	3.72	9.05	<0.001	0.64
		F	104	13.3	4.26	11.0	4.37	−2.3	3.85	6.09	<0.001	0.53
	Anxiety *	All	216	12.3	4.39	8.1	3.30	−4.2	4.09	15.19	<0.001	>1.00
		M	112	12.0	4.71	7.6	3.21	−4.4	4.33	10.74	<0.001	>1.00
		F	104	12.7	4.00	8.7	3.32	−4.1	3.84	10.78	<0.001	>1.00
	Depression *	All	216	12.6	4.91	8.1	3.90	−4.5	4.43	14.99	<0.001	>1.00
		M	112	12.3	5.14	7.6	3.73	−4.7	4.75	10.49	<0.001	>1.00
		F	104	12.9	4.65	8.6	4.03	−4.3	4.08	10.79	<0.001	0.99
	Fatigue *	All	216	14.3	4.58	9.7	4.39	−4.6	5.49	12.22	<0.001	>1.00
		M	112	14.0	4.70	8.6	4.07	−5.4	5.56	10.23	<0.001	>1.00
		F	104	14.6	4.46	10.9	4.44	−3.7	5.30	7.10	<0.001	0.83
	Sleep	All	216	10.8	2.69	10.3	1.87	−0.4	2.78	2.35	0.010	0.19
	disturbance *	M	112	11.2	2.57	10.4	2.12	−0.8	2.88	3.09	0.001	0.36
		F	104	10.3	2.75	10.3	1.57	0.0	2.62	0.07	0.470	0.01
	Social role *	All	216	14.7	4.36	11.2	4.42	−3.5	4.13	12.48	<0.001	0.80
		M	112	13.8	5.08	9.9	4.71	−3.9	4.75	8.75	<0.001	0.80
		F	104	15.7	3.17	12.6	3.60	−3.1	3.31	9.43	<0.001	0.90
	Pain	All	216	15.8	4.81	10.7	4.91	−5.1	4.68	16.00	<0.001	>1.00
	Interference *	M	112	14.7	5.54	9.3	4.93	−5.4	5.04	11.35	<0.001	>1.00
		F	104	16.9	3.56	12.2	4.44	−4.8	4.24	11.41	<0.001	>1.00
Pain (GRS)	FacePain *	All	155	2.92	1.42	1.02	1.19	−1.9	1.7	13.9	<0.001	>1.00
		M	72	2.99	1.45	0.93	1.08	−2.06	1.58	11.02	<0.001	>1.00
		F	83	2.87	1.4	1.1	1.27	−1.77	1.8	8.95	<0.001	>1.00
Pain (NRS)	Lowest pain *	All	155	5.2	2.11	2.68	2.03	−2.52	2.04	15.33	<0.001	>1.00
		M	72	5.43	2.3	2.68	2.37	−2.75	2.31	10.12	<0.001	>1.00
		F	83	5	1.93	2.69	1.7	−2.31	1.77	11.88	<0.001	>1.00
	Average pain *	All	155	7.45	1.6	4.28	1.95	−3.16	2.03	19.38	<0.001	>1.00
		M	72	7.56	1.78	3.93	2.13	−3.62	2.19	14.04	<0.001	>1.00
		F	83	7.35	1.44	4.59	1.73	−2.76	1.8	13.98	<0.001	>1.00
	Strongest pain*	All	155	8.34	1.5	5.38	2.37	−2.95	2.53	14.52	<0.001	>1.00
		M	72	8.47	1.57	5	2.71	−3.47	2.8	10.51	<0.001	>1.00
		F	83	8.22	1.44	5.71	1.98	−2.51	2.19	10.41	<0.001	>1.00
	Pain reduction	All	155	37.42	24.7	59.81	23.64	22.39	28.1	9.92	<0.001	0.93
	(%)	M	72	35	26.48	61.11	23.59	26.11	30.51	7.26	<0.001	>1.00
		F	83	39.52	23	58.67	23.78	19.16	25.58	6.82	<0.001	0.82
HADS	Anxiety *	All	8	13.4	2.00	7.3	3.45	−6.1	4.58	3.78	0.003	>1.00
		M	4	14.0	2.00	4.8	0.50	−9.3	2.50	7.40	0.003	>1.00
		F	4	12.8	2.06	9.8	3.30	−3.0	4.08	1.47	0.119	>1.00
	Depression *	All	8	15.0	3.30	8.4	7.29	−6.6	7.01	2.67	0.016	>1.00
		M	4	14.3	3.10	2.8	1.26	−11.5	3.70	6.22	0.004	>1.00
		F	4	15.8	3.77	14.0	6.16	−1.8	6.13	0.57	0.304	0.34
MSSS	Stiffness *	All	17	2.2	0.81	2.0	1.98	−0.2	1.25	0.78	0.225	0.16
		M	9	2.4	0.97	2.1	2.21	−0.2	1.30	0.51	0.311	0.13
		F	8	2.1	0.61	1.9	1.82	−0.3	1.28	0.55	0.299	0.18
	Spasticity *	All	17	1.9	0.50	0.4	0.70	−1.5	0.62	10.10	<0.001	>1.00
		M	9	2.0	0.60	0.4	0.83	−1.6	0.73	6.42	<0.001	>1.00
		F	8	1.9	0.38	0.4	0.57	−1.5	0.53	7.94	<0.001	>1.00
	Walking ability *	All	17	2.9	0.94	2.3	1.74	−0.7	1.00	2.68	0.008	0.46
		M	9	2.7	1.18	2.1	2.28	−0.6	1.24	1.35	0.107	0.31
		F	8	3.2	0.52	2.4	0.96	−0.8	0.71	3.00	0.010	0.97
	Body movement *	All	17	2.7	0.96	1.9	1.40	−0.8	0.83	3.79	0.001	0.64
		M	9	2.5	1.17	1.8	1.86	−0.7	1.12	1.79	0.056	0.43
		F	8	2.8	0.70	1.9	0.73	−0.9	0.35	7.00	<0.001	>1.00
MIDOS	(Global) *	All	32	15.4	7.77	5.9	3.93	−9.4	6.91	7.73	<0.001	>1.00
		M	24	13.7	7.21	5.2	3.66	−8.5	6.79	6.10	<0.001	>1.00
		F	8	20.5	7.50	8.1	4.16	−12.4	6.84	5.11	0.001	>1.00

*M* = mean, *SD* = standard deviation, *n* = count, *T* = *t*-test statistic, *p* = value of *p* of the *t*-test, *d* = effect size Cohen’s d for the t-test with d = 0.2, 0.5 and 0.8 indicating small, moderate and large effect sizes. HADS = Hospital Anxiety and Depression Scale, MIDOS = Minimal Documentation System on Distressing Symptoms, MSSS = Multiple Sclerosis Spasticity Scale, PROMIS = Patient Reported Outcome Measurement Information System, TSQM II = Treatment Satisfaction Questionnaire for Medication (version II), WHO-5 = 5-item World Health Organization Well-Being Index, GRS = Graphical Rating Scale, NRS = Numerical Rating Scale.

* indicate parameters for which an amelioration of the symptoms are represented by a negative number for the changes in column 9. Note that *d*-values are limited here to a maximum of 1.0 in order to avoid over-interpretation of the results.

Satisfaction with the treatment (measured by the TSQM II, Figure 2C) increased in the three subscales perceived effectiveness, side effects and overall satisfaction, while the ease of taking medication (“convenience”) rose (all with large effect sizes and *p* < 0.001).

In accordance with this, well-being (as measured by the WHO-5, Figure 2D) increased from 6.0 ± 4.6 to 13.8 ± 5.8 out of 25 points (d > 1.0; p < 0.001).

In the PROMIS (*cf.* Figure 2E and Table 2) participants reported having fewer problems with their bodily functions (physical functioning). They experienced less anxious and depressive feelings and their fatigue decreased as well. Similarly, they reported fewer problems fulfilling their social roles and the pain was perceived to have less impact on their daily lives (pain interference scale). The only exception to the large perceived effects found in this questionnaire was sleep disturbance, which showed only a small improvement over time.

*N* = 155 (72%) participants indicated that pain relief was the primary reason for their cannabinoid prescriptions. These were asked in greater detail about their perceived pain intensities. On a 6-point face pain scale average pain intensity decreased from 2.9 ± 1.4 to 1.0 ± 1.2 (*d* > 1.0, *p* < 0.001). In addition, pain was rated on an 11-point numerical rating scale from 0 to 10 (NRS) (Figure 2F) for the most severe, average, and least severe pain intensity during the day, and had decreased in all three (*d* > 1.0, *p* < 0.001). Finally, the pain reduction experienced through the daily medication (on a scale of 0 to 100) increased from 37.4 ± 24.7 to 59.8 ± 23.6 (d = 0.93, *p* < 0.001).

Eight participants who reported anxiety or depression as their primary health problem completed the HADS questionnaire described substantial reductions of both anxiety (HADS-A) and depressive moods (HADS-D), thus changing from classified as “severe (case)” (scores >10.5) to “moderate (borderline case)” (scores between 7.5 and 10.5) or even “inconspicuous (non-case)” (scores ≤7.5).

In *n* = 17 participants with multiple sclerosis, the MSSS-88 was used. Self-ratings for muscle stiffness decreased with low effect size, whereas scores for spasticity, impairment of walking ability, and impairment of body mobility decreased with moderate to large effect sizes (*p* < 0.001).

Thirty-two patients with weight loss without anorectic symptoms completed the MIDOS questionnaire. The global symptoms’ sum scale (ranging from 0 to 30) decreased from 15.4 ± 7.8 to 5.9 ± 3.9 (*d* > 1.0, *p* < 0.001), indicating clinically significant amelioration of symptoms.

Since only *n* = 4 participants with ADHD symptoms participated in the study, their results in the ASRS are not presented here to avoid over-interpretation.

None of the participants filled out the ANIS questionnaire for anorexia nervosa.

### 3.8 Confounder analysis

We tested whether putative confounders could explain the observed effects for the TSQM II total score, WHO-5 and pain intensity (average daily pain intensity). None of the tested confounders (sex; age; income: household income; prior attitude toward cannabinoid therapy; prior experience with cannabis/cannabinoids; treatment duration in months; initiative to commence the cannabinoid therapy; insurance: statutory vs. private; reimbursement: cannabinoids paid directly by insurer, reimbursed or paid out of pocket; the kind of cannabinoid preparation; traumatic experiences; and additional therapies) could contribute any significant explanation to the observed changes in the TSQM II total score (*cf.* Table 3). Eta-squared values for treatment duration (0.150), the presence of additional therapies in general (0.093) and psychotherapy in particular (0.062) indicate a strong contribution of these factors to the observed changes of the WHO-5 score, while eta-squared values for income (0.096), treatment duration (0.151), reimbursement of costs (0.082), the kind of the cannabinoid preparation (0.066) and the presence of additional therapies (0.064) contributed to the explanation of the average daily pain intensity. While all these contributions have a *p* < 0.01 and the eta-squared values indicate medium to large effects, they explanatory value is limited though, as the eta-squared value of the main within-factor (time) is much higher with values between 0.59 and 0.64.

**Table 3:** Tests for the contribution of putative confounders on the score differences (changes) between the time of the survey and the self-recalled time before the commencement of the cannabinoid therapy.

		*F*-value for factor			Partial eta2 for factor		
Target	Confounder	Time	Confounder	Interaction	Time	Confounder	Interaction
Global TSQM II Score	–	372.68			0.6342		
	Sex	373.10	1.15	1.24	0.6355	0.0053	0.0058
	Age	402.75	2.11	6.78	0.6551	0.0290	0.0876
	Income	369.12	1.18	0.66	0.6385	0.0327	0.0185
	Prior attitude	376.80	0.34	1.59	0.6410	0.0064	0.0293
	Prior experience	372.83	1.86	1.09	0.6353	0.0086	0.0051
	Treatm duration	387.96	0.59	1.42	0.6666	0.0598	0.1332
	Initiative	369.83	1.27	0.59	0.6367	0.0234	0.0110
	Insurance	376.29	0.59	2.04	0.6385	0.0056	0.0188
	Reimbursement	373.81	0.97	1.22	0.6381	0.0135	0.0169
	Cannabinoid	385.48	2.09	8.38	0.6430	0.0097	0.0377
	Trauma Experience	373.86	1.87	1.34	0.6371	0.0173	0.0124
	Additional Therapies	370.98	3.62	0.02	0.6342	0.0166	0.0001
	Physical therapy	373.83	0.47	1.66	0.6360	0.0022	0.0077
	Surgical therapy	371.07	1.81	0.07	0.6342	0.0084	0.0003
	Analgesics	371.65	0.03	0.40	0.6346	0.0002	0.0019
	Opioids	373.61	0.20	1.54	0.6358	0.0009	0.0071
	Anti-depressants	371.88	0.05	0.54	0.6347	0.0002	0.0025
	Psychotherapy	373.84	0.30	1.67	0.6360	0.0014	0.0077
WHO-5	–	379.07			0.6381		
	Sex	385.32	7.41	4.55	0.6429	0.0335	0.0208
	Age	390.66	3.62	3.19	0.6482	0.0487	0.0432
	Income	380.05	2.05	1.09	0.6452	0.0556	0.0304
	Prior attitude	385.16	1.74	1.86	0.6461	0.0320	0.0341
	Prior experience	377.57	6.43	0.15	0.6383	0.0292	0.0007
	Treatm Duration	403.42	1.63	1.66	0.6753	0.1500	0.1521
	Initiative	381.15	1.34	1.30	0.6437	0.0247	0.0240
	Insurance	379.97	1.13	1.26	0.6408	0.0105	0.0117
	Reimbursement	388.24	3.31	2.73	0.6468	0.0448	0.0372
	Cannabinoid	385.92	4.32	4.89	0.6433	0.0198	0.0223
	Trauma Experience	376.32	2.81	0.22	0.6386	0.0257	0.0021
	Additional Therapies	385.01	22.01	4.37	0.6427	0.0933	0.0200
	Physical therapy	381.64	3.86	2.46	0.6407	0.0177	0.0113
	Surgical therapy	377.56	0.11	0.14	0.6382	0.0005	0.0007
	Analgesics	400.97	3.39	13.42	0.6520	0.0156	0.0590
	Opioids	382.88	3.31	3.16	0.6415	0.0153	0.0145
	Anti-depressants	382.96	9.16	3.20	0.6415	0.0411	0.0148
	Psychotherapy	378.23	14.05	0.52	0.6387	0.0616	0.0024
Average Pain	–	259.89			0.5855		
	Sex	273.59	3.48	10.70	0.5992	0.0187	0.0552
	Age	266.05	2.61	2.45	0.5951	0.0415	0.0391
	Income	258.39	3.14	0.82	0.5921	0.0958	0.0270
	Prior attitude	259.45	0.99	0.92	0.5904	0.0214	0.0201
	Prior experience	260.85	3.44	1.68	0.5877	0.0185	0.0091
	Treatm Duration	260.43	1.45	1.02	0.6136	0.1506	0.1106
	Initiative	265.71	1.25	2.03	0.5961	0.0271	0.0432
	Insurance	257.23	1.00	0.06	0.5856	0.0109	0.0006
	Reimbursement	275.25	5.36	4.63	0.6033	0.0816	0.0712
	Cannabinoid	276.13	12.83	12.50	0.6014	0.0655	0.0639
	Trauma Experience	257.71	0.09	0.23	0.5861	0.0010	0.0025
	Additional Therapies	269.28	12.49	7.65	0.5954	0.0639	0.0401
	Physical therapy	271.30	6.93	9.08	0.5972	0.0365	0.0473
	Surgical therapy	258.69	0.79	0.15	0.5857	0.0043	0.0008
	Analgesics	262.05	3.67	2.53	0.5888	0.0197	0.0136
	Opioids	268.45	9.73	7.06	0.5946	0.0505	0.0371
	Anti-depressants	263.69	8.67	3.69	0.5903	0.0452	0.0198
	Psychotherapy	258.74	1.78	0.19	0.5857	0.0096	0.0010

For each of the three target scores (global TSQM II, WHO-5 and average pain), in a first line *F*-values and partial eta2 for the ANOVA with the sole factor (time) without any confounder are given in bold. In the following ten lines, *F*-values and partial eta2 are given for the Confounder (Conf) as a second fixed factor and the interaction (Inter).

Confounders: sex; age: age group; income: household income; prior attitude toward cannabinoid therapy; prior experience with cannabis/cannabinoids; treatment duration in months; initiative to commence the cannabinoid therapy; insurance: statutory vs. private; reimbursement: cannabinoids paid directly by insurer, reimbursed or paid from patient’s own pocket; kind of cannabinoid medicine; experienced traumas; and whether and if so, which additional therapies took place. TSQM II = Treatment Satisfaction Questionnaire for Medication (version II), WHO-5 = 5-item World Health Organization Well-Being Index.

## 4 Discussion

The results of this cross-sectional study suggest that most of the surveyed outpatients treated with prescription cannabinoids in Germany subjectively experience health benefits and symptom reduction associated with these therapies.

The authors analyzed a sample of 216 datasets that were systematically collected in a way that was originally intended to provide a representative sample. The demographic characteristics of the participants were similar to those observed in the much larger national surveillance survey, with almost even gender representation and a majority of middle-aged participants. Additionally, pain was the main health concern in over 70% of participants and the spectrum of adverse events reported was similar, with frequent mild side effects and rare occurrence of serious side effects (6, 34, 35).

However, the sample differed significantly from the national surveillance survey in terms of the proportion of prescribed cannabinoids (6). The current study observed a higher proportion of medical cannabis flowers (45% vs. 16.5%), cannabis extracts (24% vs. 13%), and nabiximols (13% vs. 8%), while a lower proportion of dronabinol (20% vs. 62.2%) was reported.

The potential bias in either study, particularly in the smaller sample of the study presented here, may account for this difference. The authors hypothesize that the participants in the current study might have been more willing to report anonymously on dosage forms, such as cannabis flowers, which are more commonly associated with illegal drug use in society. It was only in 2017 that the possibility arose to prescribe medical cannabis flowers in Germany. Before that, the use of cannabis flowers was illegal in any way. Additionally, the national surveillance survey only included cannabinoid therapies reimbursed by statutory health insurers, which might explain why prescription cannabis flowers were less frequently mentioned there (1, 6, 34).

Across all diagnoses and symptom groups, participants in this sample reported positive effects on emotional states and quality of life. This suggests that a significant mediating factor may be the stress-reducing effect of cannabis-based drugs, consistent with the importance of the endocannabinoid system for stress regulation and corresponding preclinical data (36). In contrast, the opioid system appears to have more ambivalent effects on stress regulation because the kappa opioid receptor signaling pathway is activated by stress stimuli that produce both aversion and dysphoria in humans and other animal species (37). Findings in chronic non-cancer pain that opioid therapy often does not result in a satisfactory therapeutic outcome or improvement in function (38, 39) may be based on this insight and make opioids less suitable for long duration pain management.

The participants’ attitude toward cannabis and cannabinoids was neutral to slightly positive before cannabinoid therapy and only became predominantly positive during therapy, which can be understood as an indication that the concrete therapeutic experience might have contributed to the patients’ opinion formation. This may disagree with the hypothesis of other authors that cannabinoid patients are predominantly individuals who were cannabis-affine prior to starting their prescription treatment (11, 40).

The sociodemographic data in this sample and the participants’ attitudes toward cannabis suggest that cannabinoid patients in Germany do not represent a particularly unusual group compared to the general population with comparable health issues. This contradicts the idea formulated elsewhere that these patients might be a rather young and male patient clientele with pronounced previous (illegal) drug experience (40). Yet, it is noteworthy that cannabinoid therapies in Germany are more likely to reach patients with high case severity and symptom burden due to the legal framework.

Most problems encountered by the patients during the prescription process of cannabinoid therapies did not originate from physicians, but instead were primarily perceived due to reimbursement issues involving health insurance carriers. Notably, approximately 25% of participants with statutory health insurance coverage opted to pay for their cannabinoid treatments out of pocket. This is likely due to the current legal situation in Germany, where the prescription of cannabinoid medications is characterized by significant complexity and administrative hurdles, comparable to those encountered when prescribing off-label drugs, both for patients and practitioners (German Social Code (SGB), Part V, § 31, Section 6).

The identification of physicians who treat patients with cannabinoids required a substantial recruitment effort. Most physicians stated that they did not prescribe cannabinoids and therefore could not invite patients to the study. Only a few individual physicians prescribed cannabinoids and of those only 34 informed their patients about this survey meaning, all study participants were prescribed cannabinoids by one of these 34 physicians. This observation may indicate that the rather strict and time-consuming regulations on cannabinoid prescribing (compared to opioids and other medical narcotics) mean that few physicians consider cannabinoid treatments, while many others may avoid cannabinoid treatment altogether, which may also be related to other factors, such as a lack of knowledge and/or missing experience with cannabinoids, prejudice against cannabis, etc. This situation poses a potential problem as it may lead to both an overuse by those physicians who frequently prescribe cannabinoids and an underuse by other physicians, as reflected in our sample, in which only 43 of all contacted physicians prescribe cannabinoids.

Comparable studies in Germany in which patients were directly questioned about their cannabinoid therapy are rare. Both the national web-based surveillance survey (6) and a survey among pain therapists in the German federal state of Saarland only asked physicians (41). Similar to our results, chronic pain was the main indication for the use of medical cannabis in both studies. Patient-triggered motivation for cannabinoid use was even higher in the Saarland study (63%) than in our study (47%), although the dropout rate of 29.7% was independent of who initiated the therapy (41). To date, only one study in Germany has directly interviewed chronic pain patients (*n* = 187) about their experiences with cannabis-based medications (42), including interviews with their treating physicians. In this study, both patients and physicians agreed that cannabis-based medications were particularly beneficial in relieving chronic pain and improving function, with patients rating these effects higher than physicians (42). Interestingly, in a recent survey of 207 patients eligible for the Pennsylvania medical cannabis program (43), anxiety disorders were the most common underlying condition at 50.1%, followed by chronic pain (22.3%) and post-traumatic stress disorder (PTSD) (7.9%). However, approximately 68% of all patients in this study reported using medical cannabis for various pain conditions. This finding underscores the large overlap of chronic pain with mood disorders, particularly anxiety and PTSD (44), conditions in which the pleiotropic effects of medical cannabinoids may be particularly beneficial. Fittingly, a questionnaire-based cross-sectional study of 429 chronic non-cancer pain (CNCP) patients in Israel showed that quality of life improved more markedly than pain intensity with the use of medical cannabis (45).

### 4.1 Limitations

This study has several limitations that need to be considered when interpreting the results.

First, the cross-sectional study design introduces a potential for systematic recency vs. recall bias. Participants were asked to retrospectively report their self-recalled health status, symptoms, and attitudes before their cannabinoid therapy commenced. The length of the recall period varied from less than 1 year to up to 8 years, which may have distorted perceived effects and side effects and exaggerated the benefits of cannabinoid therapies. The significances and effect sizes presented in this study should be interpreted with caution due to this potentially large source of bias.

Second, to minimize selection bias, physicians were contacted in strata reflecting the proportion of the four medical specialist groups and population of the federal states. However, the survey was conducted during the SARS-CoV-2 pandemic, which may have led to fewer physicians overall making their cannabinoid patients aware of the survey. This could have resulted in an underrepresentation of patients who were treated less successfully, since prescribing physicians may have been less motivated to invite them to participate. It is not possible to specify or even roughly estimate from the survey data how many patients were actually approached by their physicians. Additionally, slow recruitment and time restrictions led to deviations from the original recruitment strategy in Brandenburg, which reduces the informative value of the data set.

Third, additional selection bias may have occurred at the study population level. Patients who were approached to participate in the survey may have been successfully treated and more inclined to participate than those who were unsuccessfully treated or experienced more side effects or drug interactions of cannabinoids.

Fourth, expectation bias on the part of patients could have influenced the results. The high access barriers for reimbursable cannabinoid therapies in Germany mean that most participants in this study are chronically ill individuals with a high burden of disease who likely have high expectations of cannabinoid therapies. This could have biased the dataset toward a favorable evaluation of prescription cannabinoid therapies.

Fifth, the relatively small number of survey participants limits the informative value of the dataset. This is even more the case as – due to the anonymity – we do not have any hint as to the reasons why some participants stopped participating directly after starting the survey.

Sixth, the web-based-only survey nature of the study may have led to additional bias. The survey may have attracted an above-average number of technology or internet-competent individuals, possibly leading to an underrepresentation of older cannabinoid patients. However, the similarities in age and gender between the samples of this study and the national surveillance survey (6, 34) as well as the typical proportion between privately and publicly insured patients rather indicates a sample that is not too much biased in this direction.

Seventh, the anonymous participation mode may have introduced a possible bias, as some surveys may have been filled out entirely or partially not by patients but by partners, family members, or other persons. However, this possibility of influence appears to be rather theoretical against the background of the methodology described (13).

Eighth, several additional minor limitations of this study have been described in the protocol publication (13).

Finally, this uncontrolled survey study is a cross-sectional observational study of evidence class IV, which means that no causal conclusions can be drawn from the results. This is a fundamental limitation of any such study designs (especially as they become vulnerable to all analyzed possible confounders), and of particular relevance for this study regarding a one-time survey, in which the participants’ subjective current health status was compared with their self-recalled health status before the start of cannabinoid therapy in the same session. Therefore, comparative data from this exploratory dataset must be interpreted with caution and all due restraint.

## 5 Conclusion

In this cross-sectional exploratory study, participants were web-surveyed about their experiences with outpatient cannabinoid treatments. Most of the surveyed participants found therapy with cannabinoids to be effective compared to their treatment prior to medical cannabinoid prescription use. In Germany, due to regulatory barriers, this therapeutic option is mainly available for patients with a considerable case severity and disease burden. Numerous limitations are associated with the design of this cross-sectional study and restrict the informative value of this class IV evidence dataset; in particular, no conclusive statements on causality can be made. This observational study nevertheless provides starting points for further discussion in the context of planning clinical cannabinoid trials and formulating appropriate research questions, involving the patients’ perspectives. Also, due to lack of high-quality Ia and Ib evidence regarding the clinical use of cannabinoids in most medical indications, well-designed RCTs are warranted for the further development of this emerging field in medicine.

## Data availability statement

The raw data supporting the conclusions of this article will be made available by the authors, without undue reservation.

## Ethics statement

The studies involving humans were approved by Charité – Universitätsmedizin Berlin, Berlin, Germany EA no.: EA1/327/20. The studies were conducted in accordance with the local legislation and institutional requirements. The participants provided their written informed consent to participate in this study.

## Author contributions

JF, MK, and CK: conceptualization and methodology. JF, FIK, and FF: formal analysis. JF, FIK, MK, and FF: validation. JF, MK, FF, and CK: investigation. MK, AM, and CK: resources. JF, FIK, EH, LZ, and FF: data curation. JF and FIK: writing – initial draft. JF, FIK, EK, LZ, MJ, FK, FF, VM, EH, CW, AM, MK, and CK: writing – review & editing. JF, MK, AM, and CK: supervision and project administration. CK: funding acquisition. All authors read and approved the final manuscript.
